# Daily dietary patterns associated with *Helicobacter pylori* infection and eradication-related adverse events, but not eradication rate: a cross-sectional and prospective cohort study

**DOI:** 10.3389/fnut.2026.1821481

**Published:** 2026-08-04

**Authors:** Huan Zhang, Qimeng Pang, Nannan Li, Jumei Yin, Jing Zhang, Xiaojing Zhu, Lei Shang, Yongquan Shi, Zheyi Han

**Affiliations:** 1 State Key Laboratory of Holistic Integrative Management of Gastrointestinal Cancers and National Clinical Research Center for Digestive Diseases, Xijing Hospital of Digestive Diseases, Fourth Military Medical University, Xi’an, China; 2 Department of Gastroenterology, The 961th Hospital of Joint Logistics Support Force of PLA, Qiqihar, China; 3 Department of Gastroenterology, 3201 Hospital, Hanzhong, Shaanxi, China; 4 Department of Health Statistics, School of Preventive Medicine, Fourth Military Medical University, Xi'an, China; 5 Department of Gastroenterology, Air Force Medical Center, Fourth Military Medical University, Beijing, China

**Keywords:** adverse events, dietary patterns, eradication, *Helicobacter pylori*, infection

## Abstract

**Background:**

Daily dietary patterns are associated with *Helicobacter pylori* (Hp) infection, though with notable regional differences. However, their impact on Hp eradication efficacy and treatment-related adverse events remains unclear.

**Methods:**

This two-part observational study assessed the link between diet and Hp. A cross-sectional analysis (*n* = 464 after matching) identified dietary patterns associated with infection, and a prospective cohort (*n* = 476) evaluated how these patterns influenced eradication rates and adverse events. Dietary patterns were derived via exploratory factor analysis (EFA), with logistic regression and restricted cubic splines used to analyze associations.

**Results:**

In the cross-sectional phase, a “traditional northwest” pattern increased infection risk (OR = 3.776, 95% CI 2.099–6.792), while a “plant-based” pattern reduced it (OR = 0.479, 95% CI 0.268–0.856). In the prospective cohort, the overall eradication rate was 88.7%. None of the identified dietary patterns (high-protein, traditional northwest, or healthy) showed a significant association with eradication rates. However, adverse events occurred in 20.4% of participants during eradication therapy, with bitter taste being most common (32.0%). The traditional northwest pattern, characterized by high-salt diet, was associated with a reduced risk of these events (OR = 0.396, 95% CI 0.188–0.837).

**Conclusion:**

Daily dietary patterns were associated with Hp infection and eradication-related adverse events, but not eradication rate. These findings suggest that dietary patterns may be applicable to Hp prevention and management strategies, independent of their influence on eradication efficacy.

## 1 Introduction

*Helicobacter pylori* (Hp) infection remains a significant global public health concern. Although advances in public health measures and optimized clinical regimens have led to a declining infection rate worldwide, rising antibiotic resistance and suboptimal treatment adherence continue to pose serious challenges to Hp control (1, 2). Environmental factors substantially influence both Hp susceptibility and pathogenicity; among these, diet occupies a particularly important role given its direct contact with the gastric mucosa, through which it modulates bacterial virulence, disease progression, and host immune responses (3, 4).

Early nutritional epidemiology focused on individual foods or nutrients, with vitamin C-rich fruits and vegetables, garlic, honey, and green tea proposed as protective factors (5–7), while high-salt diets and processed meats may increase infection risk (8, 9). However, single-food approaches fail to capture the complexity of dietary interactions, which has prompted the adoption of dietary pattern analysis as a more holistic means of assessing the overall relationship between diet and Hp infection (3). Studies have shown that high-carbohydrate and sweet food patterns are positively associated with Hp infection, whereas patterns characterized by higher intakes of organ meats, fish, and poultry are inversely associated (8). Notably, dietary patterns are highly region- and culture-specific, limiting cross-regional generalizability of factor or principal component analysis-derived patterns (10). Within China, studies on dietary patterns and Hp infection remain limited and concentrated in eastern coastal regions such as Zhejiang and Tianjin (8, 11). Given the distinctive dietary culture of Northwest China, systematically investigating its characteristic patterns and their relationship with Hp infection is essential for advancing regional epidemiological understanding.

Furthermore, as antibiotic resistance continues to rise (12) and patient adherence to multi-drug combination regimens declines (13), adjunctive therapies and dietary interventions in Hp management have attracted growing attention. Dietary interventions offer several potential advantages, including improving patient adherence, reducing costs, and preserving gut microbiota balance. Nevertheless, most existing studies focus on the adjunctive effects of specific bioactive components during eradication therapy (6, 14), leaving a gap in systematic evidence regarding how habitual dietary patterns affect eradication efficacy and the incidence of treatment-related adverse events. A clearer understanding of the role that dietary patterns play during Hp eradication therapy could not only enable more personalized dietary guidance for patients, but may also offer novel strategies to enhance eradication success rates and reduce adverse events.

In light of these considerations, this study aims to: (1) identify and characterize the major dietary patterns in a Northwest Chinese population and examine their associations with Hp infection through a cross-sectional analysis; and (2) investigate the influence of habitual dietary patterns on the efficacy of Hp eradication therapy and the occurrence of treatment-related adverse events through a prospective cohort design—with the overarching goal of providing scientific evidence to inform comprehensive Hp prevention and control strategies.

## 2 Methods

### 2.1 Study subjects

This study comprised two parts. The first was a single-center cross-sectional study enrolling outpatients from the Department of Gastroenterology, Xijing Hospital (October 2023–October 2024), who underwent ^13^C/^14^C urea breath test, rapid urease test, or fecal Hp antigen test for Hp diagnosis. The second part was a non-randomized, single-center, prospective cohort study. Asymptomatic Hp-positive carriers (confirmed by any of the above three assays) were enrolled from the same department during the same period. Of 548 treatment-naive Hp-infected patients recruited, 44 were lost to follow-up, 22 withdrew, and those with missing or invalid dietary data were excluded, yielding a final analytical sample of 476 patients (Supplementary Figure 1).

This study was approved by the Ethics Committee of Xijing Hospital, Fourth Military Medical University (No. KY20232384-C-1).

### 2.2 Inclusion criteria and exclusion criteria

*Inclusion criteria*: (1) Aged 18–70 years, regardless of gender; (2) Definite Hp test results (including ^13^C/^14^C urea breath test, rapid urease test, and fecal Hp antigen test); (3) Willingness to receive Hp eradication therapy among Hp-positive participants. Hp eradication therapy was administered in accordance with the regimens recommended by Chinese clinical guidelines.

*Exclusion criteria*: (1) A history of definite Hp infection with prior antibiotic-based eradication therapy; (2) Severe organ damage or complications (e.g., liver cirrhosis, uremia, etc.); (3) Continuous use of antiulcer drugs, antibiotics, or bismuth-containing compounds (administered at least within 2 weeks prior to Hp testing); (4) Pregnant or lactating women; (5) History of upper gastrointestinal surgery; (6) History of malignant tumors; (7) Presence of mental disorders; (8) Special dietary habits (e.g., caloric restriction, vegetarianism); (9) Refusal to sign the informed consent form.

### 2.3 Sample size calculation

For the cross-sectional section, based on a 50% Hp prevalence in Shaanxi (15), with *α* = 0.05 and precision = 0.1, PASS calculation yielded a sample size of 402. Accounting for 10% invalid questionnaires, at least 447 participants were required.

The empirical method was used in the prospective cohort study. Factor analysis required a sample size of 10–15 times the number of factors, a total of 28 factors, and at least 420 participants were included. According to the loss to follow-up rate of 10%, 467 participants were ultimately required.

### 2.4 Data collection

#### 2.4.1 Demographic data

Demographic data included age, gender, height, weight, educational level (High school or below/College or above), occupation (Indoor worker/Outdoor worker), marital status (Married/Other), and number of household members (1/2/3/4/≥5).

#### 2.4.2 Lifestyle and medical history

Demographic and lifestyle characteristics included periodontal disease history (Yes/No), dental caries (Yes/No), tooth loss (Yes/No), dentures (Yes/No), drinking water source (Tap water/Non-tap water/Both), raw water consumption (Yes/No), eating out (Frequently/Occasionally/Never), dining environment (Good/Poor), handwashing before meals (Always/Frequently/Occasionally/Never), handwashing after using the toilet (Always/Frequently/Occasionally/Never), taste preference (Salty-preferring/Moderate/Light), shared mouthwash cup with others (Frequently/Occasionally/Never), and shared drinking cup with others (Frequently/Occasionally/Never).

#### 2.4.3 Dietary survey

A food frequency questionnaire (FFQ) developed and validated in Shaanxi Province, China (16) was used. Participants were asked to report both the frequency of food intake and the consumption amount for each food item. The intake of most foods was quantified using the Chinese traditional weight unit “liang” (equivalent to 50 grams). This FFQ included 27 common food items, which were categorized into nine groups based on intake frequency. Daily intake (in grams) of each food group was calculated by integrating the reported intake frequency and portion size provided by participants, and these data were used to derive dietary patterns.

#### 2.4.4 Assessment of adverse events

Treatment-related adverse events were assessed based on participants’ self-reports during follow-up after eradication therapy. Participants were asked whether they had experienced any discomfort or symptoms after initiating treatment, including gastrointestinal symptoms and other treatment-related complaints.

### 2.5 Statistical analysis

Statistical analyses were performed with SPSS 26.0 (IBM Corp., USA). Continuous data were expressed as mean ± SD or median (IQR) based on normality; categorical data as *n* (%). Independent samples *t*-test and *χ*^2^ test were used for between-group comparisons of continuous and categorical data, respectively. Dietary patterns were identified by exploratory factor analysis (EFA), retaining factors with eigenvalue > 1 (scree plot-confirmed) and applying varimax rotation. Patterns were named according to food groups with loadings > 0.25. Because the cross-sectional and prospective cohort analyses involved different analytic populations and outcomes, EFA was performed separately for each component of the study. Logistic regression was used for multivariate analysis, with results reported as ORs (95% CIs). Restricted cubic spline (RCS) regression was applied to examine potential non-linear dose–response relationships between dietary pattern scores and adverse event risk during Hp eradication therapy. Statistical significance was set at two-tailed *p* < 0.05.

## 3 Results

### 3.1 Dietary patterns and Hp infection (cross-sectional study)

#### 3.1.1 Basic characteristics

A total of 873 participants were initially enrolled, with baseline characteristics stratified by Hp infection status provided in Supplementary Table 1. The median age was 47.4 years, and the overall Hp positivity rate was 79.8%.

Due to uneven sample distribution, 1:2 propensity score matching (PSM) was performed (caliper = 0.05) with all baseline variables as covariates. Post-matching, 464 participants were included (298 Hp-positive vs. 166Hp-negative), with no significant differences in baseline characteristics between groups.

#### 3.1.2 Dietary patterns

After varimax rotation, EFA identified three dietary patterns. Key factor loadings of foods for each pattern are presented in Supplementary Table 2. These patterns collectively explained 26.7% of the total variance. Based on their variance contribution, they were defined as follows: Factor 1 (Traditional Northwest Pattern), Factor 2 (Balanced Pattern), and Factor 3 (Plant-Based Pattern).

#### 3.1.3 Association between dietary patterns and Hp infection rate

Table 1 presents associations between dietary patterns and Hp infection. The Traditional Northwest Pattern was positively associated with Hp infection risk (Q4 vs. Q1: OR = 3.776, 95% CI: 2.099–6.792), while the Plant-Based Pattern was inversely associated (Q4 vs. Q1: OR = 0.479, 95% CI: 0.268–0.856). The Balanced Pattern showed a non-significant trend toward reduced risk. These associations remained consistent after covariate adjustment.

**Table 1:** Association between dietary patterns and *Helicobacter pylori* infection rate.

Dietary pattern	Quartiles of dietary pattern score [OR (95% CI)]			
	Q1	Q2	Q3	Q4
Traditional Northwest pattern				
Model 1	1.0 (Reference)	1.639 (0.950, 2.828)	3.425 (1.905, 6.158)*	3.776 (2.099, 6.792)*
Model 2	1.0 (Reference)	1.765 (0.986, 3.161)	3.803 (2.037, 7.097)*	4.628 (2.361, 9.069)*
Balanced pattern				
Model 1	1.0 (Reference)	1.202 (0.668, 2.161)	1.104 (0.618, 1.971)	0.848 (0.480, 1.498)
Model 2	1.0 (Reference)	1.313 (0.706, 2.443)	1.162 (0.613, 2.200)	0.857 (0.451, 1.630)
Plant-based pattern				
Model 1	1.0 (Reference)	1.172 (0.646, 2.126)	0.791 (0.443, 1.412)	0.479 (0.268, 0.856)*
Model 2	1.0 (Reference)	1.165 (0.629, 2.155)	0.824 (0.452, 1.502)	0.457 (0.249, 0.837)*

Model 1: Unadjusted; Model 2: Adjusted for age, gender, BMI, educational level, number of household members, marital status, occupation, dining environment, eating out frequency, periodontal disease history, dental caries, tooth loss, dentures, drinking water source, raw water consumption, handwashing frequency before meals and after using the toilet, taste preference, and shared use of mouthwash cups and drinking cups. *Q*, quartile; *p < 0.05.

### 3.2 Dietary patterns and Hp eradication (cohort study)

#### 3.2.1 Baseline characteristics

A total of 476 participants were enrolled in this study. Supplementary Table 3 presents the baseline data of patients with successful and failed Hp eradication, with an overall eradication rate of 88.7%. Univariate analysis revealed no significant differences in baseline characteristics between the two groups.

#### 3.2.2 Dietary patterns

After varimax rotation, EFA identified three dietary patterns, with primary food factor loadings presented in Table 2. These patterns collectively explained 26.0% of the total variance. Defined based on their variance contribution: Factor 1 (High-Protein Pattern): Characterized by high intake of red meat (0.681), poultry (0.680), beverages (0.596), animal offal (0.494), rice (0.434), eggs (0.358), low-fat milk (0.310), aquatic products (0.342), processed meat (0.276), and fresh milk (0.253). Factor 2 (Traditional Northwest Pattern): Dominated by pickled foods (0.675), beer (0.467), tea (0.460), liquor (0.437), processed meat (0.375) and noodles (0.327). Factor 3 (Healthy Pattern): Marked by abundant consumption of soy products (0.572), cruciferous vegetables (0.564), fungi (0.523), fresh fruits (0.465), nuts (0.445), green vegetables (0.404), aquatic products (0.377), tubers (0.377), and eggs (0.268).

**Table 2:** Factor loadings of food intake patterns.

Food group	Factor 1: High-protein pattern	Factor 2: Traditional Northwest pattern	Factor 3: Healthy pattern
Red meat (pork, beef, lamb, etc.)	**0.681**	0.148	0.014
Poultry (chicken, duck, etc.)	**0.680**	0.047	0.070
Beverages	**0.596**	0.032	**−0.235**
Animal offal	**0.494**	0.241	0.138
Rice	**0.434**	0.136	0.076
Eggs	**0.358**	**−0.306**	**0.268**
Low-fat milk	**0.310**	**−0.257**	0.204
Steamed bun/steamed stuffed bun/pancake	**−0.263**	0.085	0.247
Rice wine	0.159	0.069	−0.132
Salted vegetables/salted mustard/salted eggs/sufu (fermented tofu)	0.124	**0.675**	0.085
Pickled vegetables/sour vegetable soup (jiangshui cai)/kimchi	−0.035	**0.654**	0.048
Beer	0.111	**0.467**	−0.147
Tea	0.116	**0.460**	0.134
Liquor	0.011	**0.437**	−0.083
Sausage/ham/bacon/smoked meat	**0.276**	**0.375**	0.104
Noodles	**−0.279**	**0.327**	0.108
Fresh milk	**0.253**	**−0.319**	0.148
Rice noodles (mipi/mixian)	0.170	0.237	0.115
Red wine	0.045	0.207	−0.011
Soy products (including soybean, tofu, yuba, vegetarian chicken, soymilk, etc.)	0.081	0.060	**0.572**
Cruciferous vegetables (including radish, Chinese cabbage, broccoli, cauliflower, cabbage, etc.)	−0.062	0.003	**0.564**
Fungi (including tremella, shiitake, needle mushroom, portobello, etc.)	0.098	0.030	**0.523**
Fresh fruits	0.201	−0.201	**0.465**
Nuts (including peanut, melon seed, walnut, almond, etc.)	−0.043	0.080	**0.445**
Green vegetables (green vegetable, rape, spinach, water spinach, lettuce, etc.)	0.038	−0.148	**0.404**
Aquatic products (fish, shrimp, etc.)	**0.342**	0.101	**0.377**
Tubers (including potato, sweet potato, Chinese yam, lotus root, etc.)	−0.149	0.201	**0.332**
Eigenvalue	2.873	2.257	1.886
Explained variance (%)	10.641	8.360	6.985
Cumulative variance (%)	10.641	19.001	25.986

Factor loadings with absolute values > 0.25 are presented. Values with absolute values > 0.25 are shown in bold.

#### 3.2.3 Basic characteristics stratified by dietary patterns

Participants were categorized into quartiles of dietary pattern scores for baseline comparison (Supplementary Tables 4–6). In the High-Protein Pattern, Q4 individuals were significantly younger, more likely male, better educated, less likely married, more frequently working indoors, having better dining environments, eating out more often, preferring salty tastes, and washing hands more frequently before meals and after toilet use (all *p* < 0.05). In the Traditional Northwest Pattern, Q4 individuals were more likely male, working indoors, having larger households, being smokers, having higher BMI, poorer dining environments, preferring salty tastes, consuming more non-tap water, washing hands less frequently after toilet use, and sharing drinking cups more often (all p < 0.05).

#### 3.2.4 Association between dietary patterns and Hp eradication rate

The association between dietary patterns and Hp eradication rate is presented in Table 3. Independent variables were quartiles of the identified dietary pattern factor scores, with the first quartile (Q1) as the reference group for each pattern. Results showed no statistically significant associations between the High-Protein Pattern, Traditional Northwest Pattern, or Healthy Pattern and Hp eradication rate.

**Table 3:** Association between dietary patterns and *Helicobacter pylori* eradication rate.

Dietary pattern	Quartiles of dietary pattern scores [OR (95%CI)]			
	Q1	Q2	Q3	Q4
High-protein pattern				
Model 1	1.0 (Reference)	1.101 (0.486, 2.949)	0.960 (0.431, 2.138)	1.068 (0.469, 2.432)
Model 2	1.0 (Reference)	1.018 (0.414, 2.504)	0.848 (0.339, 2.121)	0.859 (0.311, 2.371)
Traditional Northwest pattern				
Model 1	1.0 (Reference)	0.858 (0.362, 2.032)	0.742 (0.321, 1.717)	0.710 (0.306, 1.644)
Model 2	1.0 (Reference)	0.839 (0.328, 2.144)	0.614 (0.237, 1.595)	0.577 (0.208, 1.603)
Healthy pattern				
Model 1	1.0 (Reference)	0.797 (0.380, 1.673)	1.739 (0.726, 4.167)	1.285 (0.567, 2.909)
Model 2	1.0 (Reference)	0.658 (0.292, 1.481)	1.637 (0.642, 4.172)	1.179 (0.491, 2.830)

Model 1: Unadjusted; Model 2: Adjusted for age, gender, BMI, educational level, number of household members, marital status, occupation, smoking status, dining method, dining environment, periodontal disease history, dental caries, tooth loss, dentures, drinking water source, raw water consumption, handwashing frequency before meals and after using the toilet, taste preference, and shared use of mouthwash cups and drinking cups. *Q*, quartile; *p < 0.05.

In addition, subgroup analyses stratified by age and sex were performed (Supplementary Table 7). After adjusting for all covariates, no statistically significant associations were observed between the High-Protein Pattern, Traditional Northwest Pattern, or Healthy Pattern and Hp eradication rate in any subgroup.

### 3.3 Dietary patterns and adverse reactions of Hp eradication therapy

#### 3.3.1 Baseline characteristics stratified by adverse events

**Figure 1:**
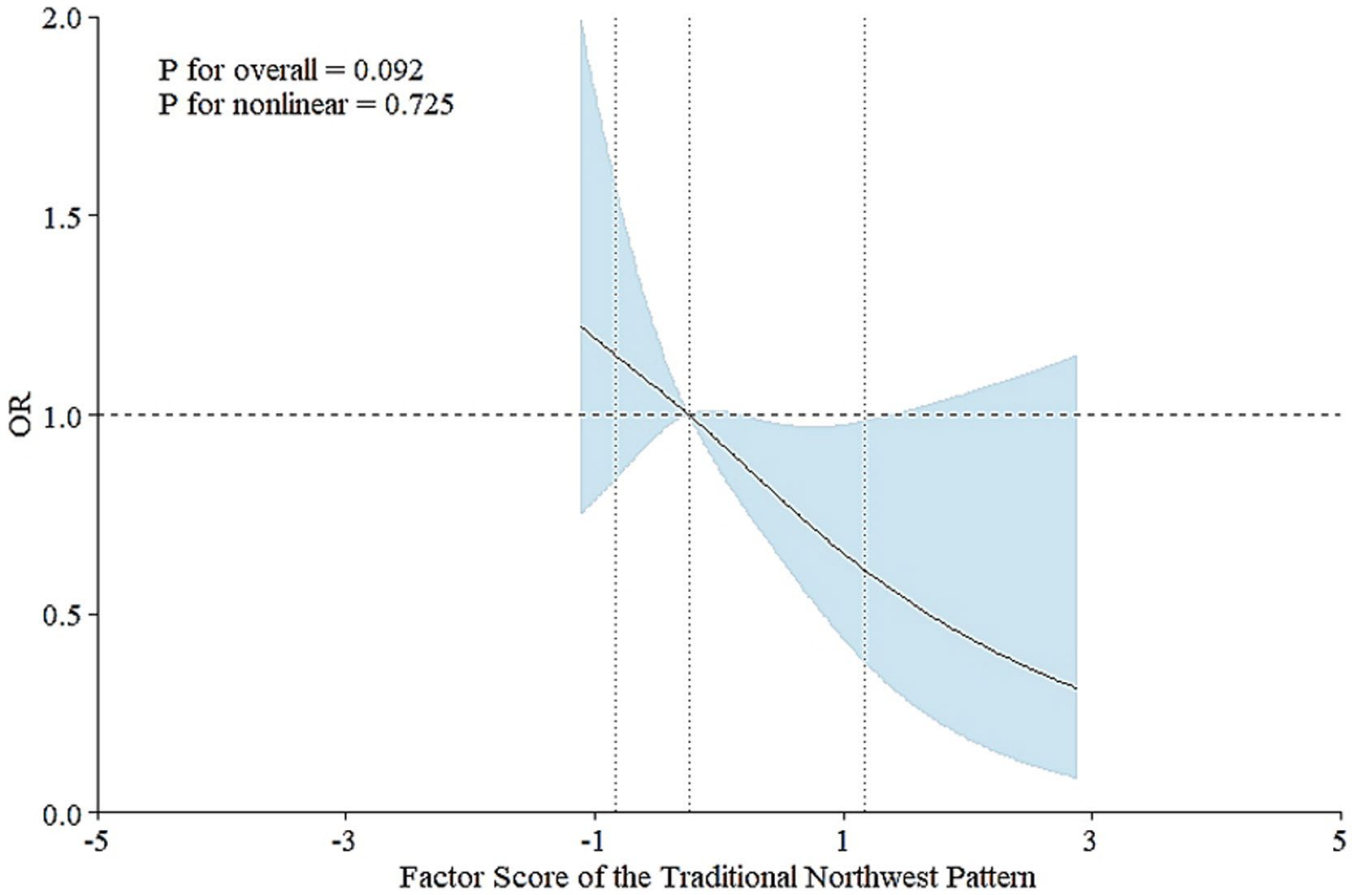
Distribution of adverse events among participants.

Adverse events occurred in 20.4% of participants (97/476; Figure 1), most commonly bitter taste (32.0%), nausea (30.9%), diarrhea (18.6%), and dizziness (16.5%). Less frequent events included abdominal distension (10.3%), anorexia (9.3%), vomiting and abdominal pain (4.1% each), fatigue (3.1%), skin rash (2.1%), and headache, foot swelling, and insomnia (1.0% each).

Baseline comparison revealed that participants with adverse events were significantly younger (median 44.0 vs. 50.0 years, *p* = 0.003), more likely non-smokers (84.5% vs. 70.4%) with fewer current smokers (9.3% vs. 21.9%, *p* = 0.013), and had a lower rate of dentures use (10.3% vs. 22.7%, *p* = 0.007) compared to those without adverse events (Supplementary Table 8). No significant differences were found in other baseline variables (all *p* > 0.05).

#### 3.3.2 Association between adverse reactions to Hp eradication therapy and dietary patterns

Table 4 presents associations between dietary patterns and adverse events. Only the Traditional Northwest Pattern showed a significant association, with Q4 linked to a reduced risk of adverse events compared to Q1 (OR = 0.396, 95% CI: 0.188–0.837, *p* < 0.05). No significant associations were observed for the High-Protein or Healthy patterns.

**Table 4:** Association between dietary patterns and overall adverse events.

Dietary pattern	Quartiles of dietary pattern scores [OR (95%CI)]			
	Q1	Q2	Q3	Q4
High-protein pattern	1.0 (Reference)	0.984 (0.505, 1.917)	1.073 (0.543, 2.120)	0.969 (0.473, 1.982)
Traditional Northwest pattern	1.0 (Reference)	0.921 (0.483, 1.756)	1.244 (0.662, 2.338)	0.396 (0.188, 0.837)*
Healthy pattern	1.0 (Reference)	1.074 (0.561, 2.058)	1.218 (0.639, 2.323)	0.953 (0.483, 1.881)

Model: Adjusted for age, smoking status and dentures. *Q*, quartile; *p < 0.05.

**Figure 2:**
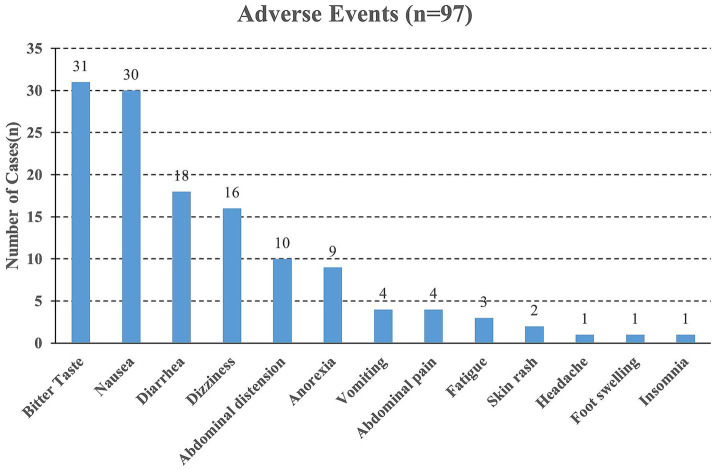
RCS plot of the association between traditional Northwest dietary pattern score and adverse events.

RCS analysis (Figure 2) revealed a gradual downward trend in adverse event risk with increasing Traditional Northwest Pattern scores, though statistical significance was not reached (overall *p* = 0.092, P for nonlinearity = 0.725), consistent with the protective trend observed in Q4.

## 4 Discussion

This study employed a cross-sectional analysis and a prospective cohort to examine associations between dietary patterns and Hp infection prevalence, eradication success, and treatment-related adverse events in Northwest China. In the cross-sectional analysis, the Plant-Based Pattern was inversely associated with Hp infection, while the Traditional Northwest Pattern (high-salt, strong-flavored) was positively associated. In the prospective cohort, dietary patterns were not significantly associated with eradication success; however, Q4 of the Traditional Northwest Pattern was associated with significantly lower adverse event risk compared to Q1 after covariate adjustment. RCS analysis showed a decreasing trend in adverse event risk with increasing Traditional Northwest Pattern scores, though without reaching full significance (*p* = 0.09). These findings suggest that dietary patterns may influence Hp infection epidemiology and treatment tolerance, rather than eradication efficacy per se.

The associations between Hp and different dietary patterns observed in this study are consistent with previous reports, supporting the reliability of our data and methods (11). Diet and Hp jointly contribute to reshaping the gastric microenvironment (17, 18). Research suggests human infection with Hp dates back at least 100,000 years, making the bacterium anatomically contemporary with modern humans (19). Under long-term evolutionary pressure, both factors have profoundly influenced gastric selective pressures and adaptive changes. Hp can induce inflammation by activating the IL-6/STAT3 pathway, enrich specific pathogens (e.g., *Streptococcus*, *Fusobacterium*), and promote the production of carcinogenic metabolites such as N-nitroso compounds (20). High-salt or high-fat diets can drive similar adverse alterations in the gastric environment through these same pathways; thus, a synergistic or interactive effect between the two is biologically plausible (4).

The prospective cohort component of this study found no significant association between habitual dietary patterns and Hp eradication rates. One plausible explanation is the potent efficacy of multi-antibiotic therapeutic regimens, which likely masks any subtle, long-term physiological modulatory effects of dietary patterns on the gastric environment or Hp viability (21). In other words, the diet-shaped gastric environment can be readily disrupted by antibiotics during treatment. The gastrointestinal microbiota provides a useful illustration of this phenomenon: gut microbiota diversity typically declines markedly in the short-term following eradication therapy, with restoration to baseline levels requiring anywhere from 6 months to 2 years (22, 23). Indeed, certain commonly consumed foods and their bioactive components may enhance eradication therapy outcomes, such as bee products, dairy products, vegetables, fruits, and spices (6, 14). The actual efficacy of this approach is dose-dependent, with purified bioactive components typically being those capable of significantly improving eradication rates, a standard that ordinary dietary foods rarely meet. For instance, probiotic preparations have been reported to increase Hp eradication rates of standard regimens by approximately 5–15% (24). However, aside from fermented dairy products, the viable microbial content in other everyday foods ranges between 10^4^ and 10^7^ CFU/g or even lower, substantially below the microbial levels contained in probiotic preparations (10^9^–10^12^ CFU/g) (25). Similarly, studies indicate that vitamin C can enhance the efficacy of standard triple therapy (26). The recommended intake of vitamin C for healthy adults ranges from 95 to 110 mg/day (27), and vitamin C intake among plant-based diet populations can even reach 213 mg/day (28), this still falls far short of the supplemental doses used in RCTs (1,000 mg/day) (26, 29). For Hp eradication, pharmacological intervention remains the decisive factor. Future research should prioritize extracts of bioactive components from anti-Hp foods or plants, as daily dietary intake is unlikely to reach therapeutic doses.

Although dietary patterns were unrelated to therapeutic efficacy, interestingly, we found that the Traditional Northwest pattern, characterized by high salt intake, was associated with a reduced risk of adverse effects. The occurrence of adverse events represents a critical reason for decreased adherence to multi-antibiotic eradication regimens and subsequent treatment failure, thus this finding provides novel insights for optimizing Hp management. Fermented foods such as pickled and preserved vegetables constitute the predominant factors in the traditional Northwestern pattern, and such probiotic-rich foods resembling Korean kimchi may help alleviate Hp-associated gastritis (30). Long-term consumption of fermented foods may confer greater “resilience” to the gut microbiota, rendering it less susceptible to severe dysbiosis under antibiotic assault, thereby reducing the likelihood of gastrointestinal discomfort (31–33). Regional spices and spicy condiments prevalent in the Northwest diet (e.g., capsaicin in chili peppers) can stimulate sensory neurons in the gastric mucosa, leading to increased mucosal blood flow and enhanced secretion of mucus and bicarbonate (34). This may provide a “buffer” that increases gastric tolerance to high-dose antibiotic irritation, an effect that remains unaffected by eradication therapy (35). Bitter taste was the most reported adverse effect, and the high sodium salt content in the Traditional Northwest pattern is recognized as an effective bitterness inhibitor (36), which may reduce the perception of treatment-induced bitterness and thus mitigate adverse effects. Meanwhile, studies have demonstrated a significant positive correlation between salty taste perception threshold and bitter taste perception threshold (37). This implies that individuals insensitive to salty taste (i.e., consuming saltier foods) are typically also insensitive to bitter taste. Chinese people traditionally maintain a bland diet during illness, which aligns with the core Traditional Chinese Medicine theory from the Huangdi Neijing that “the spleen and stomach constitute the foundation of postnatal life” (38). While this tradition holds merit, our study suggests that maintaining certain dietary flavors, at least during Hp eradication therapy, may actually help alleviate treatment-related adverse effects.

The inverse association between a high-salt diet and adverse events should be interpreted with caution. Experimental studies provide a possible mechanism, but not definitive evidence. Takeuchi et al. (39) showed that pre-exposure to mildly hypertonic NaCl, such as 1 mol/L NaCl, increased endogenous prostacyclin release and protected the rat gastric mucosa against subsequent ethanol injury through sensory nerve activation. Another study demonstrated that mild high-salt stimulation (5% NaCl) could also induce adaptive cytoprotection through mechanisms partly independent of prostaglandin synthesis, by increasing gastric mucus, enhancing gastric secretion, and strengthening the physical barrier of the gastric mucosa (40). Repeated aspirin administration enhanced the defensive capacity of the rat gastric mucosa against subsequent exposure to various strong irritants (41), but diet-related preconditioning effects derive from acute local stimulation models, and their long-term protective effects in humans remain unproven. Chronic high-salt intake unequivocally damages the gastric mucosa and drives Hp adaptation to the hyperosmotic environment, promoting the selection of strains with higher oncogenic potential (42). Consequently, our findings only support maintaining existing dietary preferences to preserve flavor during eradication therapy, rather than recommending a long-term increase in dietary salt intake, which remains an established risk factor for gastric diseases.

This study is the first to systematically investigate associations between habitual dietary patterns and both Hp infection and eradication outcomes. However, several limitations warrant consideration. First, dietary intake was assessed via FFQ, which is subject to recall bias. The FFQ was designed to capture foods commonly consumed by the local population rather than to comprehensively estimate total energy and nutrient intake. This highly customized questionnaire may effectively reflect habitual food preferences among populations in Northwestern China; however, its simplicity may introduce some measurement error in assessing overall dietary diversity. Second, despite instructions for participants to maintain habitual eating patterns during eradication therapy, the absence of repeated dietary assessments raises the possibility of additional bias resulting from unplanned dietary variations. Third, although standard first-line bismuth-containing quadruple therapy was predominantly prescribed, precise records of individual regimen modifications due to drug allergies were unavailable, potentially confounding the observed adverse event profiles. Fourth, residual confounding from unmeasured variables cannot be excluded despite comprehensive covariate adjustment. Importantly, we lacked data on key microbiological and clinical determinants of eradication success, including antibiotic resistance profiles, *H. pylori* virulence factors (e.g., CagA and VacA), and initial bacterial load. Since antibiotic resistance is the primary driver of treatment failure, and virulence factors as well as bacterial load strongly modulate host immune responses and drug efficacy, the omission of these parameters could confound or mask any subtle influence of diet on eradication rates. Finally, the relatively high Hp eradication rate in this study, together with the limited sample size, may have reduced statistical power and constrained our ability to conduct more detailed subgroup analyses. Larger-scale, multi-center studies across diverse populations are needed to validate these findings and explore region-specific dietary influences.

## 5 Conclusion

In summary, plant-based dietary patterns are associated with lower Hp infection rates, whereas the traditional Northwestern dietary pattern is associated with increased infection risk. The traditional Northwestern dietary pattern may reduce the risk of adverse events during eradication therapy rather than directly affecting eradication rates. This study provides novel insights for Hp prevention and management, suggesting that nutritional research aimed at improving Hp eradication rates should focus more on bioactive component extracts from foods rather than specific foods or overall dietary patterns.

## Data Availability

The original contributions presented in the study are included in the article/Supplementary material, further inquiries can be directed to the corresponding authors.
