# Supplementary material for: Daily dietary patterns associated with Helicobacter pylori infection and eradication-related adverse events, but not eradication rate: a cross-sectional and prospective cohort study
Source: Front Nutr. 2026 Aug 4;13:1821481. doi: 10.3389/fnut.2026.1821481 (PMC13481178; doi:10.3389/fnut.2026.1821481)
Supplement: Supplementary file 1 [file Table_1.DOCX]

S Figure 1. Flow chart of cohort study section.

| **S Table 1. Comparison of baseline characteristics between Hp negative and Hp positive participants.** | | | | | | |
| --- | --- | --- | --- | --- | --- | --- |
|  | Before PSM | | *P* value | After PSM | | *P* value |
|  | Hp-(176) | Hp+(697) |  | Hp-(166) | Hp+(298) |  |
| **Age (year)** | 51.0(37.3,56.0) | 49.0(35.0,58.0) | 0.637 | 51.0(38.0,56.0) | 49.0(36.8,57.0) | 0.582 |
| **Gender,[n(%)]** | | | 0.117 |  |  | 0.815 |
| Male | 79(44.9) | 359(51.5) |  | 75(45.2) | 138(46.3) |  |
| Female | 97(55.1) | 338(48.5) |  | 91(54.8) | 160(53.7) |  |
| **Educational level ,[n(%)]** | | | 0.019 |  |  | 0.803 |
| High school or below | 115(65.3) | 387(55.5) |  | 105(63.3) | 185(62.1) |  |
| College or above | 61(34.7) | 310(44.5) |  | 61(36.7) | 113(37.9) |  |
| **Marital status,[n(%)]** | | | 0.311 |  |  | 0.817 |
| Married | 157(89.2) | 599(85.9) |  | 149(89.8) | 264(88.6) |  |
| Other | 19(10.8) | 98(14.1) |  | 17(10.2) | 34(11.4) |  |
| **Occupation,[n(%)]** | | |  |  |  | 0.855 |
| Outdoor worker | 36(20.5) | 167(24.0) | 0.377 | 35(21.1) | 65(21.8) |  |
| Indoor worker | 140(79.5) | 530(76.0) |  | 131(78.9) | 233(78.2) |  |
| **Number of household members ,[n(%)]** | | | 0.111 |  |  | 0.998 |
| 1 | 5(2.8) | 12(1.7) |  | 5(3.0) | 8(2.7) |  |
| 2 | 21(11.9) | 82(11.8) |  | 21(12.7) | 36(12.1) |  |
| 3 | 44(25.0) | 181(26.0) |  | 41(24.7) | 77(25.8) |  |
| 4 | 67(38.1) | 208(29.8) |  | 60(36.1) | 108(36.2) |  |
| ≥5 | 39(22.) | 214(30.7) |  | 39(23.5) | 69(23.2) |  |
| **BMI(kg/m2)** | 22.3(20.0,24.5) | 22.6(20.6,24.6) | 0.078 | 22.1±3.2 | 22.3±3.2 | 0.482 |
| **Dining environment ,[n(%)]** | | | 0.607 |  |  | 1.000 |
| Poor | 7(4.0) | 20(2.9) |  | 5(3.0) | 9(3.0) |  |
| Good | 169(96.0) | 677(97.1) |  | 161(97.0) | 289(97.0) |  |
| **Eating out ,[n(%)]** | | | 0.015 |  |  | 0.334 |
| Never | 18(10.2) | 40(5.7) |  | 17(10.2) | 23(7.7) |  |
| Occasionally | 38(21.6) | 211(30.3) |  | 34(20.5) | 77(25.8) |  |
| Frequently | 120(68.2) | 446(64.0) |  | 115(69.3) | 198(66.4) |  |
| **Taste preference ,[n(%)]** | | | <0.001 |  |  | 0.362 |
| Light | 88(50.0) | 175(25.1) |  | 80(48.2) | 124(41.6) |  |
| Moderate | 61(34.7) | 360(51.6) |  | 59(35.5) | 115(38.6) |  |
| Salty-preferring | 27(15.3) | 162(23.2) |  | 27(16.3) | 59(19.8) |  |
| **Periodontal disease history, [n(%)]** | | | 0.027 |  |  | 0.499 |
| No | 140(79.5) | 601(86.2) |  | 134(80.7) | 248(83.2) |  |
| Yes | 36(20.5) | 96(13.8) |  | 32(19.3) | 50(16.8) |  |
| **Dental caries ,[n(%)]** | | | 0.987 |  |  | 0.517 |
| No | 136(77.3) | 539(77.3) |  | 126(75.9) | 234(78.5) |  |
| Yes | 40(22.7) | 158(22.7) |  | 40(24.1) | 64(21.5) |  |
| **Tooth loss ,[n(%)]** | | | 0.141 |  |  | 0.826 |
| No | 130(73.9) | 553(79.3) |  | 123(74.1) | 218(73.2) |  |
| Yes | 46(26.1) | 144(20.7) |  | 43(25.9) | 80(26.8) |  |
| **Dentures,[n(%)]** | | | 0.852 |  |  | 0.898 |
| No | 143(81.3) | 562(80.6) |  | 134(80.7) | 242(81.2) |  |
| Yes | 33(18.8) | 135(19.4) |  | 32(19.3) | 56(18.8) |  |
| **Drinking water source ,[n(%)]** | | | <0.001 |  |  | 0.989 |
| Tap water | 130(73.9) | 413(59.3) |  | 125(75.3) | 225(75.5) |  |
| Non-tap water | 11(6.3) | 11(1.6) |  | 6(3.6) | 10(3.4) |  |
| Both | 35(19.9) | 273(39.2) |  | 35(21.1) | 63(21.1) |  |
| **Raw water consumption ,[n(%)]** | | | 0.297 |  |  | 0.839 |
| No | 167(94.9) | 643(92.3) |  | 157(94.6) | 279(93.6) |  |
| Yes | 9(5.1) | 54(7.7) |  | 9(5.4) | 19(6.4) |  |
| **Handwashing before meals ,[n(%)]** | | | 0.003 |  |  | 0.994 |
| Never | 0(0.0) | 8(1.1) |  | 0(0.0) | 0(0.0) |  |
| Occasionally | 128(72.7) | 431(61.8) |  | 119(71.7) | 215(72.1) |  |
| Frequently | 10(5.7) | 105(15.1) |  | 9(5.4) | 16(5.4) |  |
| Always | 38(21.6) | 153(22.0) |  | 38(22.9) | 67(22.5) |  |
| **Handwashing after using the toilet ,[n(%)]** | | | 0.002 |  |  | 0.989 |
| Never | 0(0.0) | 1(0.1) |  | 0(0.0) | 0(0.0) |  |
| Occasionally | 128(72.7) | 428(61.4) |  | 120(72.3) | 217(72.8) |  |
| Frequently | 3(1.7) | 66(9.5) |  | 3(1.8) | 5(1.7) |  |
| Always | 45(25.6) | 202(29.0) |  | 43(25.9) | 76(25.5) |  |
| **Shared mouthwash cup with others ,[n(%)]** | | | 0.044 |  |  | 0.979 |
| Never | 157(89.2) | 571(81.9) |  | 147(88.6) | 264(88.6) |  |
| Occasionally | 17(9.7) | 99(14.2) |  | 17(10.2) | 31(10.4) |  |
| Frequently | 2(1.1) | 27(3.9) |  | 2(1.2) | 3(1.0) |  |
| **Shared drinking cup with others,[n(%)]** | | | 0.078 |  |  | 0.831 |
| Never | 167(94.9) | 635(91.1) |  | 157(94.6) | 283(95.0) |  |
| Occasionally | 9(5.1) | 44(6.3) |  | 9(5.4) | 15(5.0) |  |
| Frequently | 0(0.0) | 18(2.6) |  | 0(0.0) | 0(0.0) |  |

| **S Table 2. Factor Loadings of Food Intake Patterns** | | | |
| --- | --- | --- | --- |
| Food Group | Factor 1: Traditional Northwest Pattern | Factor 2: Balanced Pattern | Factor 3: Plant-Based Pattern |
| Pickled vegetables/salted mustard/salted eggs/sufu (fermented tofu) | **0.675** | -0.173 | 0.219 |
| Liquor | **0.659** | -0.019 | -0.102 |
| Pickled vegetables/sour vegetable soup (jiangshui cai)/kimchi | **0.636** | -0.220 | 0.228 |
| Beer | **0.609** | -0.038 | -0.110 |
| Sausage/ham/bacon/smoked meat | **0.518** | **0.310** | 0.092 |
| Tea | **0.423** | 0.183 | -0.097 |
| Red wine | **0.381** | 0.111 | -0.045 |
| Rice wine | **0.252** | 0.057 | -0.013 |
| Poultry | 0.228 | **0.625** | 0.030 |
| Red meat (pork/beef/lamb) | 0.127 | **0.601** | 0.205 |
| Fungi (including tremella, shiitake, needle mushroom, portobello, etc.) | -0.025 | **0.544** | **0.355** |
| Aquatic products (fish, shrimp, etc.) | 0.126 | **0.541** | 0.091 |
| Animal offal | **0.334** | **0.473** | -0.154 |
| Fresh fruits | -0.110 | **0.401** | **0.282** |
| Rice | 0.066 | **0.365** | -0.031 |
| Eggs | -0.063 | **0.343** | -0.037 |
| Noodles | 0.135 | **-0.339** | **0.337** |
| Beverages | 0.225 | **0.324** | 0.138 |
| Fresh milk | -0.194 | 0.245 | 0.052 |
| Rice noodles (mipi/mixian) | 0.135 | 0.201 | -0.098 |
| Nuts (including peanut, melon seed, walnut, almond, etc.) | -0.055 | 0.174 | 0.069 |
| Green vegetables (green vegetable, rape, spinach, water spinach, lettuce, etc.) | -0.202 | 0.144 | **0.613** |
| Cruciferous vegetables (including radish, Chinese cabbage, broccoli, cauliflower, cabbage, etc.) | -0.096 | 0.171 | **0.578** |
| Soy products (including soy bean, tofu, yuba, vegetarian chicken, soymilk, etc.) | -0.067 | 0.155 | **0.461** |
| Steamed bun/steamed stuffed bun/pancake | 0.051 | -0.151 | **0.423** |
| Tubers (including potato, sweet potato, Chinese yam, lotus root, etc.) | 0.079 | 0.008 | **0.330** |
| Low-fat milk | 0.006 | 0.157 | 0.182 |
| Eigenvalue | 3.119 | 2.522 | 1.579 |
| Explained variance (%) | 11.551 | 9.342 | 5.849 |
| Cumulative variance (%) | 11.551 | 20.893 | 26.742 |
| Note: Factor loadings with absolute values > 0.25 are presented. | | | |

| **S Table 3. Comparison of Baseline Characteristics Between Patients with Failed and Successful Helicobacter pylori Eradication.** | | | |
| --- | --- | --- | --- |
|  | **Eradication Failure (n=54)** | **Eradication Success (n=422)** | ***P* value** |
| **Age (year)** | 49.5(38.8,58.0) | 49.0(35.8,59.0) | 0.683 |
| **Gender,[n(%)]** |  |  | 0.458 |
| Male | 25(46.3) | 218(51.7) |  |
| Female | 29(53.7) | 204(48.3) |  |
| **Educational level ,[n(%)]** | |  | 0.832 |
| High school or below | 30(55.6) | 228(54.0) |  |
| College or above | 24(44.4) | 194(46.0) |  |
| **Marital status,[n(%)]** | |  | 0.238 |
| Married | 50(92.6) | 367(87.0) |  |
| Other | 4(7.4) | 55(13.0) |  |
| **Occupation,[n(%)]** | |  | 0.617 |
| Outdoor worker | 15(27.8) | 104(24.6) |  |
| Indoor worker | 39(72.2) | 318(75.4) |  |
| **Number of household members ,[n(%)]** | |  | 0.116 |
| 1 | 0(0.0) | 6(1.4) |  |
| 2 | 6(11.1) | 55(13.0) |  |
| 3 | 8(14.8) | 113(26.8) |  |
| 4 | 23(42.6) | 122(28.9) |  |
| ≥5 | 17(31.5) | 126(29.9) |  |
| **Smoking status, [n(%)]** | |  | 0.858 |
| Non-smoker | 40(74.1) | 309(73.2) |  |
| Ex-smoker | 3(5.6) | 32(7.6) |  |
| Current smoker | 11(20.4) | 81(19.2) |  |
| **BMI (kg/m^2^)** | 23.1±3.1 | 22.7±3.4 | 0.442 |
| **Dining method, [n(%)]** | |  | 0.581 |
| Separate dining | 4(7.4) | 46(10.9) |  |
| Communal dining | 50(92.6) | 376(89.1) |  |
| **Dining environment ,[n(%)]** | |  | 0.607 |
| Poor | 0(0.0) | 9(2.1) |  |
| Good | 54(100.0) | 413(97.9) |  |
| **Eating out ,[n(%)]** | |  | 0.887 |
| Never | 3(5.6) | 19(4.5) |  |
| Occasionally | 35(64.8) | 286(67.8) |  |
| Frequently | 16(29.6) | 117(27.7) |  |
| **Taste preference ,[n(%)]** | |  | 0.872 |
| Light | 13(24.1) | 111(26.3) |  |
| Moderate | 27(50.0) | 214(50.7) |  |
| Salty-preferring | 14(25.9) | 97(23.0) |  |
| **Periodontal disease history, [n(%)]** | |  | 0.781 |
| No | 47(87.0) | 377(89.3) |  |
| Yes | 7(13.0) | 45(10.7) |  |
| **Dental caries ,[n(%)]** | |  | 0.852 |
| No | 43(79.6) | 326(77.3) |  |
| Yes | 11(20.4) | 96(22.7) |  |
| **Tooth loss ,[n(%)]** | |  | 0.915 |
| No | 43(79.6) | 343(81.3) |  |
| Yes | 11(20.4) | 79(18.7) |  |
| **Dentures,[n(%)]** |  |  | 0.114 |
| No | 48(88.9) | 332(78.7) |  |
| Yes | 6(11.1) | 90(21.3) |  |
| **Drinking water source ,[n(%)]** | |  | 0.495 |
| Tap water | 32(59.3) | 235(55.7) |  |
| Non-tap water | 0(0.0) | 10(2.4) |  |
| Both | 22(40.7) | 177(41.9) |  |
| **Raw water consumption ,[n(%)]** | |  | 0.633 |
| No | 52(96.3) | 395(93.6) |  |
| Yes | 2(3.7) | 27(6.4) |  |
| **Handwashing before meals ,[n(%)]** | |  | 0.491 |
| Never | 1(1.9) | 7(1.7) |  |
| Occasionally | 6(11.1) | 67(15.9) |  |
| Frequently | 38(70.4) | 252(59.7) |  |
| Always | 9(16.7) | 96(22.7) |  |
| **Handwashing after using the toilet ,[n(%)]** | |  | 0.914 |
| Never | 0(0.0) | 1(0.2) |  |
| Occasionally | 5(9.3) | 40(9.5) |  |
| Frequently | 31(57.4) | 258(61.1) |  |
| Always | 18(33.3) | 123(29.1) |  |
| **Shared drinking cup with others,[n(%)]** | |  | 0.822 |
| Never | 44(81.5) | 349(82.7) |  |
| Occasionally | 7(13.0) | 57(13.5) |  |
| Frequently | 3(5.6) | 16(3.8) |  |
| **Shared mouthwash cup with others ,[n(%)]** | |  | 0.140 |
| Never | 45(83.3) | 389(92.2) |  |
| Occasionally | 6(11.1) | 23(5.5) |  |
| Frequently | 2(5.6) | 10(2.3) |  |
| **Adverse reactions, [n(%)]** | |  | 0.721 |
| None | 42(77.8) | 337(79.9) |  |
| Present | 12(22.2) | 85(20.1) |  |

| **S Table 4. Baseline Characteristics Stratified by Quartiles of High-Protein Pattern Scores.** | | | | | |
| --- | --- | --- | --- | --- | --- |
| **Variable** | **Quartiles of High-Protein Pattern Scores** | | | | ***P* value** |
|  | **Q1** | **Q2** | **Q3** | **Q4** |  |
| **Age (year)** | 55.0 (44.0,60.0) | 52.0 (39.0,60.0) | 47.0 (35.0,56.0) | 38.0 (31.0,52.0) | ＜0.001 |
| **Gender,[n(%)]** |  |  |  |  | 0.025 |
| Male | 54(45.4) | 56(47.1) | 58(48.7) | 75(63.0) |  |
| Female | 65(54.6) | 63(52.9) | 61(51.3) | 44(37.0) |  |
| **Educational level ,[n(%)]** | |  |  |  | ＜0.001 |
| High school or below | 95(79.8) | 72(60.5) | 53(44.5) | 38(31.9) |  |
| College or above | 24(20.2) | 47(39.5) | 66(55.5) | 81(68.1) |  |
| **Marital status,[n(%)]** | |  |  |  | ＜0.001 |
| Married | 111(93.3) | 112(94.1) | 104(87.4) | 90(75.6) |  |
| Other | 8(6.7) | 7(5.9) | 15(12.6) | 29(24.4) |  |
| **Occupation,[n(%)]** | |  |  |  | ＜0.001 |
| Outdoor worker | 50(42.0) | 32(26.9) | 19(16.0) | 18(15.1) |  |
| Indoor worker | 69(58.0) | 87(73.1) | 100(84.0) | 101(84.9) |  |
| **Number of household members ,[n(%)]** | | |  |  | 0.063 |
| 1 | 2(1.7) | 2(1.7) | 0(0.0) | 2(1.7) |  |
| 2 | 11(9.2) | 20(16.8) | 16(13.4) | 14(11.8) |  |
| 3 | 26(21.8) | 20(16.8) | 32(26.9) | 43(36.1) |  |
| 4 | 35(29.4) | 38(31.9) | 39(32.8) | 33(27.7) |  |
| ≥5 | 45(37.8) | 39(32.8) | 32(26.9) | 27(22.7) |  |
| **Smoking status, [n(%)]** | |  |  |  | 0.849 |
| Non-smoker | 89(74.8) | 89(74.8) | 89(74.8) | 82(68.9) |  |
| Ex-smoker | 7(5.9) | 7(5.9) | 10(8.4) | 11(9.2) |  |
| Current smoker | 23(19.3) | 23(19.3) | 20(16.8) | 26(21.8) |  |
| **BMI (kg/m^2^)** | 22.4±3.0 | 23.0±3.2 | 22.5±3.5 | 23.2±3.8 | 0.223 |
| **Dining method, [n(%)]** | |  |  |  | 0.678 |
| Separate dining | 11(9.2) | 11(9.2) | 12(10.1) | 16(13.4) |  |
| Communal dining | 108(90.8) | 108(90.8) | 107(89.9) | 103(86.6) |  |
| **Dining environment ,[n(%)]** | |  |  |  | 0.037 |
| Poor | 6(5.0) | 1(0.8) | 1(0.8) | 1(0.8) |  |
| Good | 113(95.0) | 118(99.2) | 118(99.2) | 118(99.2) |  |
| **Eating out ,[n(%)]** | |  |  |  | ＜0.001 |
| Never | 12(10.1) | 6(5.0) | 1(0.8) | 3(2.5) |  |
| Occasionally | 90(75.6) | 81(68.1) | 83(69.7) | 67(56.3) |  |
| Frequently | 17(14.3) | 32(26.9) | 35(29.4) | 49(41.2) |  |
| **Taste preference ,[n(%)]** | |  |  |  | ＜0.001 |
| Light | 35(29.4) | 39(32.8) | 37(31.1) | 13(10.9) |  |
| Moderate | 55(46.2) | 61(51.3) | 60(50.4) | 65(54.6) |  |
| Salty-preferring | 29(24.4) | 19(16.0) | 22(18.5) | 41(34.5) |  |
| **Periodontal disease history, [n(%)]** | |  |  |  | 0.875 |
| No | 106(89.1) | 108(90.8) | 106(89.1) | 104(87.4) |  |
| Yes | 13(10.9) | 11(9.2) | 13(10.9) | 15(12.6) |  |
| **Dental caries ,[n(%)]** | |  |  |  | 0.307 |
| No | 88(73.9) | 99(83.2) | 93(78.2) | 89(74.8) |  |
| Yes | 31(26.1) | 20(16.8) | 26(21.8) | 30(25.2) |  |
| **Tooth loss ,[n(%)]** | |  |  |  | 0.502 |
| No | 91(76.5) | 99(83.2) | 99(83.2) | 97(81.5) |  |
| Yes | 28(23.5) | 20(16.8) | 20(16.8) | 22(18.5) |  |
| **Dentures,[n(%)]** |  |  |  |  | 0.006 |
| No | 87(73.1) | 88(73.9) | 105(88.2) | 100(84.0) |  |
| Yes | 32(26.9) | 31(26.1) | 14(11.8) | 19(16.0) |  |
| **Drinking water source ,[n(%)]** | |  |  |  | 0.004 |
| Tap water | 81(68.1) | 74(62.2) | 61(51.3) | 51(42.9) |  |
| Non-tap water | 2(1.7) | 2(1.7) | 2(1.7) | 4(3.4) |  |
| Both | 36(30.3) | 43(36.1) | 56(47.1) | 64(53.8) |  |
| **Raw water consumption ,[n(%)]** | |  |  |  | 0.304 |
| No | 112(94.1) | 115(96.6) | 108(90.8) | 112(94.1) |  |
| Yes | 7(5.9) | 4(3.4) | 11(9.2) | 7(5.9) |  |
| **Handwashing before meals ,[n(%)]** | |  |  |  | 0.016 |
| Never | 3(2.5) | 0(0.0) | 1(0.8) | 4(3.4) |  |
| Occasionally | 15(12.6) | 24(20.2) | 17(14.3) | 17(14.3) |  |
| Frequently | 87(73.1) | 69(58.0) | 69(58.0) | 65(54.6) |  |
| Always | 14(11.8) | 26(21.8) | 32(26.9) | 33(27.7) |  |
| **Handwashing after using the toilet ,[n(%)]** | | |  |  | 0.007 |
| Never | 0(0.0) | 1(0.8) | 0(0.0) | 0(0.0) |  |
| Occasionally | 16(13.4) | 12(10.1) | 9(7.6) | 8(6.7) |  |
| Frequently | 81(68.1) | 78(65.5) | 68(57.1) | 62(52.1) |  |
| Always | 22(18.5) | 28(23.5) | 42(35.3) | 49(41.2) |  |
| **Shared drinking cup with others,[n(%)]** | | |  |  | 0.152 |
| Never | 102(85.7) | 103(86.6) | 98(82.4) | 90(75.6) |  |
| Occasionally | 16(13.4) | 11(9.2) | 16(13.4) | 21(17.6) |  |
| Frequently | 1(0.8) | 5(4.2) | 5(4.2) | 8(6.7) |  |
| **Shared mouthwash cup with others ,[n(%)]** | | |  |  | 0.927 |
| Never | 109(91.6) | 109(91.6) | 110(92.4) | 106(89.1) |  |
| Occasionally | 8(6.7) | 7(5.9) | 6(5.0) | 8(6.7) |  |
| Frequently | 2(1.7) | 3(2.5) | 3(2.5) | 5(4.2) |  |
| **Hp Eradication, [n(%)]** | |  |  |  | 0.994 |
| Failure | 14(11.8) | 13(10.9) | 14(11.8) | 13(10.9) |  |
| Success | 105(88.2) | 106(89.1) | 105(88.2) | 106(89.1) |  |

| **S Table 5.Baseline Characteristics Stratified by Quartiles of Traditional Northwest Pattern.** | | | | | |
| --- | --- | --- | --- | --- | --- |
| **Variable** | **Quartiles of Traditional Northwest Pattern Scores** | | | | ***P* value** |
|  | **Q1** | **Q2** | **Q3** | **Q4** |  |
| **Age (year)** | 52.0(36.0,60.0) | 50.0(36.0,60.0) | 47.0(35.0,58.0) | 47.0(36.0,55.0) | 0.068 |
| **Gender,[n(%)]** |  |  |  |  | ＜0.001 |
| Male | 40(33.6) | 48(40.3) | 70(58.8) | 85(71.4) |  |
| Female | 79(66.4) | 71(59.7) | 49(41.2) | 34(28.6) |  |
| **Educational level ,[n(%)]** | |  |  |  | 0.456 |
| High school or below | 57(47.9) | 66(55.5) | 67(56.3) | 68(57.1) |  |
| College or above | 62(52.1) | 53(44.5) | 52(43.7) | 51(42.9) |  |
| **Marital status,[n(%)]** | |  |  |  | 0.195 |
| Married | 104(87.4) | 108(90.8) | 98(82.4) | 107(89.9) |  |
| Other | 15(12.6) | 11(9.2) | 21(17.6) | 12(10.1) |  |
| **Occupation,[n(%)]** |  |  |  |  | ＜0.001 |
| Outdoor worker | 14(11.8) | 23(19.3) | 37(31.1) | 45(37.8) |  |
| Indoor worker | 105(88.2) | 96(80.7) | 82(68.9) | 74(62.2) |  |
| **Number of household members ,[n(%)]** | | |  |  | 0.042 |
| 1 | 4(3.4) | 0(0.0) | 2(1.7) | 0(0.0) |  |
| 2 | 23(19.3) | 17(14.3) | 11(9.2) | 10(8.4) |  |
| 3 | 28(23.5) | 32(26.9) | 31(26.1) | 30(25.2) |  |
| 4 | 30(25.2) | 39(32.8) | 31(26.1) | 42(37.8) |  |
| ≥5 | 34(28.6) | 31(26.1) | 44(37.0) | 34(28.6) |  |
| **Smoking status, [n(%)]** | |  |  |  | ＜0.001 |
| Non-smoker | 106(89.1) | 95(79.8) | 83(69.7) | 65(54.6) |  |
| Ex-smoker | 5(4.2) | 8(6.7) | 9(7.6) | 13(10.9) |  |
| Current smoker | 8(6.7) | 16(13.4) | 27(22.7) | 41(34.5) |  |
| **BMI (kg/m^2^)** | 22.0±3.6 | 22.4±3.5 | 23.1±3.2 | 23.7±3.1 | ＜0.001 |
| **Dining method, [n(%)]** | |  |  |  | 0.805 |
| Separate dining | 14(11.8) | 12(10.1) | 14(11.8) | 10(8.4) |  |
| Communal dining | 105(88.2) | 107(89.9) | 105(88.2) | 109(91.6) |  |
| **Dining environment ,[n(%)]** | |  |  |  | 0.024 |
| Poor | 0(0.0) | 0(0.0) | 4(3.4) | 5(4.2) |  |
| Good | 119(100.0) | 119(100.0) | 115(96.6) | 114(95.8) |  |
| **Eating out ,[n(%)]** |  |  |  |  | 0.076 |
| Never | 6(5.0) | 4(3.4) | 8(6.7) | 4(3.4) |  |
| Occasionally | 87(73.1) | 89(74.8) | 71(59.7) | 74(62.2) |  |
| Frequently | 26(21.8) | 26(21.8) | 40(33.6) | 41(34.5) |  |
| **Taste preference ,[n(%)]** | |  |  |  | ＜0.001 |
| Light | 46(38.7) | 34(28.6) | 26(21.8) | 18(15.1) |  |
| Moderate | 53(44.5) | 65(54.6) | 63(52.9) | 60(50.4) |  |
| Salty-preferring | 20(16.8) | 20(16.8) | 30(25.2) | 41(34.5) |  |
| **Periodontal disease history, [n(%)]** | |  |  |  | 0.523 |
| No | 103(86.6) | 104(87.4) | 109(91.6) | 108(90.8) |  |
| Yes | 16(13.4) | 15(12.6) | 10(8.4) | 11(9.2) |  |
| **Dental caries ,[n(%)]** | |  |  |  | 0.155 |
| No | 87(73.1) | 94(79.0) | 100(84.0) | 88(73.9) |  |
| Yes | 32(26.9) | 25(21.0) | 19(16.0) | 31(26.1) |  |
| **Tooth loss ,[n(%)]** |  |  |  |  | 0.713 |
| No | 93(78.2) | 100(84.0) | 97(81.5) | 96(80.7) |  |
| Yes | 26(21.8) | 19(16.0) | 22(18.5) | 23(19.3) |  |
| **Dentures,[n(%)]** |  |  |  |  | 0.357 |
| No | 94(79.0) | 95(79.8) | 101(84.9) | 90(75.6) |  |
| Yes | 25(21.0) | 24(20.2) | 18(15.1) | 29(24.4) |  |
| **Drinking water source ,[n(%)]** | |  |  |  | 0.004 |
| Tap water | 65(54.6) | 73(61.3) | 74(62.2) | 55(46.2) |  |
| Non-tap water | 7(5.9) | 0(0.0) | 1(0.8) | 2(1.7) |  |
| Both | 47(39.5) | 46(38.7) | 44(37.0) | 62(52.1) |  |
| **Raw water consumption ,[n(%)]** | |  |  |  | 0.087 |
| No | 116(97.5) | 114(95.8) | 109(91.6) | 108(90.8) |  |
| Yes | 3(2.5) | 5(4.2) | 10(8.4) | 11(9.2) |  |
| **Handwashing before meals ,[n(%)]** | |  |  |  | 0.054 |
| Never | 1(0.8) | 2(1.7) | 0(0.0) | 5(4.2) |  |
| Occasionally | 11(9.2) | 17(14.3) | 21(17.6) | 24(20.2) |  |
| Frequently | 76(63.9) | 74(62.2) | 68(57.1) | 72(60.5) |  |
| Always | 31(26.1) | 26(21.8) | 30(25.2) | 18(15.1) |  |
| **Handwashing after using the toilet ,[n(%)]** | | |  |  | 0.021 |
| Never | 1(0.8) | 0(0.0) | 0(0.0) | 0(0.0) |  |
| Occasionally | 2(1.7) | 11(9.2) | 14(11.8) | 18(15.1) |  |
| Frequently | 77(64.7) | 79(66.4) | 65(54.6) | 68(57.1) |  |
| Always | 39(32.8) | 29(24.4) | 40(33.6) | 33(27.7) |  |
| **Shared drinking cup with others,[n(%)]** | | |  |  | 0.033 |
| Never | 105(88.2) | 103(86.6) | 93(78.2) | 92(77.3) |  |
| Occasionally | 10(8.4) | 11(9.2) | 24(20.2) | 19(16.0) |  |
| Frequently | 4(3.4) | 5(4.2) | 2(1.7) | 8(6.7) |  |
| **Shared mouthwash cup with others ,[n(%)]** | | |  |  | 0.240 |
| Never | 111(93.3) | 111(93.3) | 108(90.8) | 104(87.4) |  |
| Occasionally | 6(5.0) | 4(3.4) | 10(8.4) | 9(7.6) |  |
| Frequently | 2(1.7) | 4(3.4) | 1(0.8) | 6(5.0) |  |
| **Hp Eradication, [n(%)]** | |  |  |  | 0.821 |
| Failure | 11(9.2) | 13(10.9) | 15(12.6) | 15(12.6) |  |
| Success | 108(90.8) | 106(89.1) | 104(87.4) | 104(87.4) |  |

| **S Table 6. Baseline Characteristics Stratified by Quartiles of Healthy Pattern.** | | | | | |
| --- | --- | --- | --- | --- | --- |
| **Variable** | **Quartiles of Healthy Pattern Scores** | | | | ***P* value** |
|  | **Q1** | **Q2** | **Q3** | **Q4** |  |
| **Age (year)** | 47.0(34.0,58.0) | 49.0(36.0,59.0) | 51.0(36.0,59.0) | 50.0(37.0,58.0) | 0.397 |
| **Gender,[n(%)]** |  |  |  |  | 0.999 |
| Male | 61(51.3) | 61(51.3) | 60(50.4) | 61(51.3) |  |
| Female | 58(48.7) | 58(48.7) | 59(49.6) | 58(48.7) |  |
| **Educational level ,[n(%)]** | |  |  |  | 0.433 |
| High school or below | 63(52.9) | 67(56.3) | 70(58.8) | 58(48.7) |  |
| College or above | 56(47.1) | 52(43.7) | 49(41.2) | 61(51.3) |  |
| **Marital status,[n(%)]** | |  |  |  | 0.269 |
| Married | 104(87.4) | 105(88.2) | 109(91.6) | 99(83.2) |  |
| Other | 15(12.6) | 14(11.8) | 10(8.4) | 20(16.8) |  |
| **Occupation,[n(%)]** |  |  |  |  | 0.123 |
| Outdoor worker | 32(26.9) | 33(27.7) | 34(28.6) | 20(16.8) |  |
| Indoor worker | 87(73.1) | 86(72.3) | 85(71.4) | 99(83.2) |  |
| **Number of household members ,[n(%)]** | |  |  |  | 0.326 |
| 1 | 1(0.8) | 2(1.7) | 0(0.0) | 3(2.5) |  |
| 2 | 10(8.4) | 13(10.9) | 22(18.5) | 16(13.4) |  |
| 3 | 31(26.1) | 31(26.1) | 28(23.5) | 31(26.1) |  |
| 4 | 44(37.0) | 33(27.7) | 38(31.9) | 30(25.2) |  |
| ≥5 | 33(27.7) | 40(33.6) | 31(26.1) | 39(32.8) |  |
| **Smoking status, [n(%)]** | |  |  |  | 0.170 |
| Non-smoker | 83(69.7) | 90(75.6) | 93(78.2) | 83(69.7) |  |
| Ex-smoker | 5(4.2) | 8(6.7) | 10(8.4) | 12(10.1) |  |
| Current smoker | 31(26.1) | 21(17.6) | 16(13.4) | 24(20.2) |  |
| **BMI (kg/m^2^)** | 23.1±3.3 | 22.6±3.3 | 22.5±3.9 | 22.9±3.0 | 0.473 |
| **Dining method, [n(%)]** | |  |  |  | 0.491 |
| Separate dining | 11(9.2) | 15(12.6) | 9(7.6) | 15(12.6) |  |
| Communal dining | 108(90.8) | 104(87.4) | 110(92.4) | 104(87.4) |  |
| **Dining environment ,[n(%)]** | |  |  |  | 0.952 |
| Poor | 2(1.7) | 3(2.5) | 2(1.7) | 2(1.7) |  |
| Good | 117(98.3) | 116(97.5) | 117(98.3) | 117(98.3) |  |
| **Eating out ,[n(%)]** |  |  |  |  | 0.638 |
| Never | 4(3.4) | 6(5.0) | 7(5.9) | 5(4.2) |  |
| Occasionally | 76(63.9) | 78(65.5) | 86(72.3) | 81(68.1) |  |
| Frequently | 39(32.8) | 35(29.4) | 26(21.8) | 33(27.7) |  |
| **Taste preference ,[n(%)]** | |  |  |  | 0.143 |
| Light | 26(21.8) | 38(31.9) | 30(25.2) | 30(25.2) |  |
| Moderate | 57(47.9) | 62(52.1) | 65(54.6) | 57(47.9) |  |
| Salty-preferring | 36(3.3) | 19(16.0) | 24(20.2) | 32(26.9) |  |
| **Periodontal disease history, [n(%)]** | |  |  |  | 0.834 |
| No | 104(87.4) | 107(89.9) | 108(90.8) | 105(88.2) |  |
| Yes | 15(12.6) | 12(10.1) | 11(9.2) | 14(11.8) |  |
| **Dental caries ,[n(%)]** | |  |  |  | 0.936 |
| No | 92(77.3) | 93(78.2) | 90(75.6) | 94(79.0) |  |
| Yes | 27(22.7) | 26(21.8) | 29(24.4) | 25(21.0) |  |
| **Tooth loss ,[n(%)]** |  |  |  |  | 0.920 |
| No | 98(82.4) | 95(79.8) | 95(79.8) | 98(82.4) |  |
| Yes | 21(17.6) | 24(20.2) | 24(20.2) | 21(17.6) |  |
| **Dentures,[n(%)]** |  |  |  |  | 0.576 |
| No | 98(82.4) | 91(76.5) | 93(78.2) | 98(82.4) |  |
| Yes | 21(17.6) | 28(23.5) | 26(21.8) | 21(17.6) |  |
| **Drinking water source ,[n(%)]** | |  |  |  | 0.689 |
| Tap water | 70(58.8) | 65(54.6) | 67(56.3) | 65(54.6) |  |
| Non-tap water | 0(0.0) | 3(2.5) | 3(2.5) | 4(3.4) |  |
| Both | 49(41.2) | 51(42.9) | 49(41.2) | 50(42.0) |  |
| **Raw water consumption ,[n(%)]** | |  |  |  | 0.238 |
| No | 112(94.1) | 116(97.5) | 109(91.6) | 110(92.4) |  |
| Yes | 7(5.9) | 3(2.5) | 10(8.4) | 9(7.6) |  |
| **Handwashing before meals ,[n(%)]** | |  |  |  | 0.207 |
| Never | 3(2.5) | 0(0.0) | 4(3.4) | 1(0.8) |  |
| Occasionally | 21(17.6) | 18(15.1) | 18(15.1) | 16(13.4) |  |
| Frequently | 75(63.0) | 72(60.5) | 76(63.9) | 67(56.3) |  |
| Always | 20(16.8) | 29(24.4) | 21(17.6) | 35(29.4) |  |
| **Handwashing after using the toilet ,[n(%)]** | | |  |  | 0.035 |
| Never | 0(0.0) | 0(0.0) | 1(0.8) | 0(0.0) |  |
| Occasionally | 15(12.6) | 7(5.9) | 16(13.4) | 7(5.9) |  |
| Frequently | 71(59.7) | 79(66.4) | 75(63.0) | 64(53.8) |  |
| Always | 33(27.7) | 33(27.7) | 27(22.7) | 48(40.3) |  |
| **Shared drinking cup with others,[n(%)]** | |  |  |  | 0.321 |
| Never | 92(77.3) | 104(87.4) | 96(80.7) | 101(84.9) |  |
| Occasionally | 21(17.6) | 10(8.4) | 17(14.3) | 16(13.4) |  |
| Frequently | 6(5.0) | 5(4.2) | 6(5.0) | 2(1.7) |  |
| **Shared mouthwash cup with others ,[n(%)]** | | |  |  | 0.551 |
| Never | 107(89.9) | 110(92.4) | 106(89.1) | 111(93.3) |  |
| Occasionally | 10(8.4) | 6(5.0) | 7(5.9) | 6(5.0) |  |
| Frequently | 2(1.7) | 3(2.5) | 6(5.0) | 2(1.7) |  |
| **Hp Eradication, [n(%)]** | |  |  |  | 0.289 |
| Failure | 15(12.6) | 18(15.1) | 9(7.6) | 12(10.1) |  |
| Success | 104(87.4) | 101(84.9) | 110(92.4) | 107(89.9) |  |

| **S Table 7. Subgroup Analysis of the Association Between Dietary Patterns and Helicobacter pylori Eradication Rate.** | | | | |
| --- | --- | --- | --- | --- |
|  | Quartiles of Dietary Pattern Scores [OR (95%CI)] | | | |
|  | Q1 | Q2 | Q3 | Q4 |
| High-Protein Pattern | | | | |
| Age |  |  |  |  |
| <50 years | 1.0 (Reference) | 0.323(0.101,1.034) | 0.856(0.225,3.254) | 0.878(0.227,3.393) |
| ≥50 years | 1.0 (Reference) | 2.235(0.629,7.938) | 3.166(0.841,11.920) | 1.340(0.400,4.495) |
| Gender |  |  |  |  |
| Male | 1.0 (Reference) | 1.577(0.277,8.967) | 0.596(0.141,2.511) | 0.535(0.119,2.412) |
| Female | 1.0 (Reference) | 1.014(0.306,3.355) | 1.728(0.423,7.057) | 2.294(0.443,11.871) |
| Traditional Northwest Pattern | | | | |
| Age |  |  |  |  |
| <50 years | 1.0 (Reference) | 0.310(0.072,1.328) | 0.306(0.070,1.346) | 0.314(0.069,1.421) |
| ≥50 years | 1.0 (Reference) | 2.320(0.593,9.075) | 0.933(0.278,3.123) | 0.962(0.270,3.430) |
| Gender |  |  |  |  |
| Male | 1.0 (Reference) | 1.289(0.197,8.438) | 1.002(0.186,5.387) | 0.441(0.089,2.177) |
| Female | 1.0 (Reference) | 0.442(0.125,1.563) | 0.319(0.082,1.239) | 1.726(0.258,11.541) |
| Healthy Pattern | | | | |
| Age |  |  |  |  |
| <50 years | 1.0 (Reference) | 2.397(0.549,10.464) | 1.472(0.401,5.401) | 1.158(0.322,4.163) |
| ≥50 years | 1.0 (Reference) | 0.761(0.258,2.242) | 0.654(0.193,2.209) | 2.279(0.388,13.386) |
| Gender |  |  |  |  |
| Male | 1.0 (Reference) | 1.565(0.427,5.730) | 2.257(0.540,9.436) | 2.036(0.555,7.474) |
| Female | 1.0 (Reference) | 0.382(0.106,1.370) | 2.199(0.469,10.312) | 1.270(0.288,5.598) |
| All models adjusted for age, gender, BMI, educational level, number of household members, marital status, occupation, smoking status, dining method, dining environment, periodontal disease history, dental caries, tooth loss, dentures, drinking water source, raw water consumption, handwashing frequency before meals and after using the toilet, taste preference, and shared use of mouthwash cups and drinking cups (age and gender excluded from the model when used as stratification variables). | | | | |
| Q: Quartile; *: P < 0.05 | | | | |

| **S Table 8. Baseline Characteristics Stratified by Adverse Events.** | | | |
| --- | --- | --- | --- |
|  | **No Adverse Events (n=379)** | **Adverse Events (n=97)** | ***P* value** |
| **Age (year)** | 50.0(37.0,59.0) | 44.0(33.0,55.5) | 0.003 |
| **Gender,[n(%)]** |  |  | 0.110 |
| Male | 201(53.0) | 42(43.3) |  |
| Female | 178(47.0) | 55(56.7) |  |
| **Educational level ,[n(%)]** | |  | 0.106 |
| High school or below | 213(56.2) | 45(46.4) |  |
| College or above | 166(43.8) | 52(53.6) |  |
| **Marital status,[n(%)]** | |  | 0.304 |
| Married | 335(88.4) | 82(84.5) |  |
| Other | 44(11.6) | 15(15.5) |  |
| **Occupation,[n(%)]** | |  | 0.393 |
| Outdoor worker | 98(25.9) | 21(21.6) |  |
| Indoor worker | 281(74.1) | 76(78.4) |  |
| **Number of household members ,[n(%)]** | |  | 0.647 |
| 1 | 5(1.3) | 1(1.0) |  |
| 2 | 52(13.7) | 9(9.3) |  |
| 3 | 92(24.3) | 29(29.9) |  |
| 4 | 114(30.1) | 31(32.0) |  |
| ≥5 | 116(30.6) | 27(27.8) |  |
| **Smoking status, [n(%)]** | |  | 0.013 |
| Non-smoker | 267(70.4) | 82(84.5) |  |
| Ex-smoker | 29(7.7) | 6(6.2) |  |
| Current smoker | 83(21.9) | 9(9.3) |  |
| **BMI (kg/m^2^)** | 22.8(20.9,24.8) | 22.4(20.5,24.1) | 0.177 |
| **Dining method, [n(%)]** | |  | 0.417 |
| Separate dining | 42(11.1) | 8(8.2) |  |
| Communal dining | 337(88.9) | 89(91.8) |  |
| **Dining environment ,[n(%)]** | |  | 0.330 |
| Poor | 6(1.6) | 3(3.1) |  |
| Good | 373(98.4) | 94(96.9) |  |
| **Eating out ,[n(%)]** | |  | 0.719 |
| Never | 19(5.0) | 3(3.1) |  |
| Occasionally | 255(67.3) | 66(68.0) |  |
| Frequently | 105(27.7) | 28(28.9) |  |
| **Taste preference ,[n(%)]** | |  | 0.241 |
| Light | 105(27.7) | 19(19.6) |  |
| Moderate | 186(49.1) | 55(56.7) |  |
| Salty-preferring | 88(23.2) | 23(23.7) |  |
| **Periodontal disease history, [n(%)]** | |  | 0.689 |
| No | 336(88.7) | 88(90.7) |  |
| Yes | 43(11.3) | 9(9.3) |  |
| **Dental caries ,[n(%)]** | |  | 0.068 |
| No | 301(79.4) | 68(70.1) |  |
| Yes | 78(20.6) | 29(29.9) |  |
| **Tooth loss ,[n(%)]** | |  | 0.121 |
| No | 302(79.7) | 84(86.6) |  |
| Yes | 77(20.3) | 13(13.4) |  |
| **Dentures,[n(%)]** |  |  | 0.007 |
| No | 293(77.3) | 87(89.7) |  |
| Yes | 86(22.7) | 10(10.3) |  |
| **Drinking water source ,[n(%)]** | |  | 0.102 |
| Tap water | 220(58.0) | 47(48.5) |  |
| Non-tap water | 153(40.4) | 46(47.4) |  |
| Both | 6(1.6) | 4(4.1) |  |
| **Raw water consumption ,[n(%)]** | |  | 0.966 |
| No | 356(93.9) | 91(93.8) |  |
| Yes | 23(6.1) | 6(6.2) |  |
| **Handwashing before meals ,[n(%)]** | |  | 0.152 |
| Never | 6(1.6) | 2(2.1) |  |
| Occasionally | 51(13.5) | 22(22.7) |  |
| Frequently | 236(62.3) | 54(55.7) |  |
| Always | 86(22.7) | 19(19.6) |  |
| **Handwashing after using the toilet ,[n(%)]** | |  | 0.411 |
| Never | 1(0.3) | 0(0.0) |  |
| Occasionally | 32(8.4) | 13(13.4) |  |
| Frequently | 230(60.7) | 59(60.8) |  |
| Always | 116(30.6) | 25(25.8) |  |
| **Shared drinking cup with others,[n(%)]** | |  | 0.468 |
| Never | 317(83.6) | 76(78.4) |  |
| Occasionally | 48(12.7) | 16(16.5) |  |
| Frequently | 14(3.7) | 5(5.2) |  |
| **Shared mouthwash cup with others ,[n(%)]** | |  | 0.585 |
| Never | 348(91.8) | 86(88.7) |  |
| Occasionally | 21(5.5) | 8(8.2) |  |
| Frequently | 10(2.6) | 3(3.1) |  |
